# Quantitative Cross-Cultural Similarities and Differences in Social Discounting for Gains and Losses

**DOI:** 10.3389/fpubh.2019.00297

**Published:** 2019-10-22

**Authors:** Sarah E. Stegall, Tyler Collette, Takuji Kinjo, Taiki Takahashi, Paul Romanowich

**Affiliations:** ^1^Department of Psychology, The University of Texas at San Antonio, San Antonio, TX, United States; ^2^Department of Behavioral Science, Hokkaido University, Sapporo, Japan; ^3^Department of Psychology, Gonzaga University, Spokane, WA, United States

**Keywords:** altruism, hyperbolic, power function, sign effect, exponential function, social discounting

## Abstract

Social discounting is when resource allocation decreases as social distance increases. Studies fitting different quantitative models to social discounting data have shown that a *q*-exponential function based on Tsallis' statistics best fits loss data, whereas a hyperbolic power function best fits gain data. However, a social discounting sign effect, where losses are discounted less than equivalent gains, has not been consistently found. This study fit four different quantitative social discounting models to gain and loss data for 40 United States (US) participants. We compared quantitative model fits to previous studies collected with Japanese and German participants to determine if (1) different quantitative social discounting models best fit loss and gain data, (2) US participants discounted less gains than Japanese participants, but not losses, and (3) US participants showed the sign effect. Results showed that the *q*-exponential function and the hyperbolic power function best fit median loss and gain data, respectively. There were no significant absolute differences between cultures for gains or losses, and US participants showed a robust sign effect. While most results for US participants were consistent with previous data, future cross-cultural social discounting studies are needed that manipulate sign as well as reward magnitude to determine best quantitative model fits. Social discounting results are also discussed in relation to their application to important health behaviors such as smoking and obesity.

## Introduction

Social discounting is allocating a reward across a social distance. We allocate more resources with those whom we perceive to be socially closer to us, compared to individuals who are more socially distant. Quantitatively, the results of early social discounting studies were similar to delay discounting, in that a hyperbolic function accounted for a significant amount of the obtained data (1). The hyperbolic social discounting equation is as follows:

$$\begin{array}{l} v=\;\frac{V}{1+kN}\; \end{array}$$

where *v* is the discounted value of the reward, *V* is the undiscounted value of the reward, *N* is the measure of social distance, and *k* is a constant measuring the degree of discounting. Larger *k* values indicate more discounting as a function of increasing social distance. For hyperbolic functions, value decreases quickly at smaller delays or social distances, then decreases less quickly as delays and/or social distances increase. Like previous delay discounting experiments, Jones and Rachlin (1) also showed that the hyperbolic function fit their obtained social discounting data better than an exponential function:

$$\begin{array}{l} v=Ve^{-kN}\; \end{array}$$

where the *v, V, k* and *N* parameters are the same as in the hyperbolic equation, and *e* is the base of the natural logarithm. Exponential discounting means that the discounted value decreases at a rate proportional (i.e., consistent) to the current value. Many previous human and non-human animal experiments have shown that hyperbolic functions fit obtained delay discounting data better than exponential functions, because exponential functions tend to underestimate discounting for short delays and overestimate discounting for longer delays [for review see (2)]. In terms of social discounting, exponential functions predict more sharing at closer social distances and less sharing at larger social distances than obtained by actual participant choices (1).

Two recent studies have compared quantitative social discounting models. Takahashi (3) compared social discounting rates using three different social discounting equations: the hyperbolic and exponential equations above, and a *q*-exponential discounting function based on Tsallis' statistics. The *q*-exponential equation is:

$$\begin{array}{l} v=\frac{V}{{\left(1+k\left(1-q\right)N\right)}^{1/\left(1-q\right)}}\; \end{array}$$

where *v, V, k* and *N* are the same as in the hyperbolic and exponential equations, and *q* < 1 represents the degree of “social inconsistency” from exponential discounting. When *q* approaches 1, the function is equivalent to the exponential discounting equation, and indicates that an individual is relatively consistent in their social discounting rate. Alternatively, when *q* approaches 0 the function is equivalent to hyperbolic discounting, and indicates complete inconsistency in social discounting rate. Thus, in addition to discounting rate, the *q*-exponential function provides information through the *q*-parameter about whether discounting choices are more exponential (i.e., consistent) or hyperbolic (i.e., inconsistent).

Data for both social discounting gains and losses were provided by 26 participants. Research on delay discounting provides insight on loss, showing that delays for gains are more steeply discounted than delays for losses. This is known as the sign effect [see (4)]. Social discounting for losses involves discounting adverse consequences, such as losing money, across a social distance. Takahashi (3) showed that for Japanese participants the hyperbolic equation fit the data better than the *q*-exponential equation for social discounting gains, but not for losses. The exponential equation was the worst fitting equation in both cases. Fit was determined by the Akaike Information Criterion (AIC) statistic, which calculates the tradeoff between overfitting and poor fitting models. However, the results did not show a significant sign effect, as the estimated *q*-exponential *k*-values for the median gain (*k* = 0.05) and loss (*k* = 0.06) data were similar.

Ishii and Eisen (5) also fit the three quantitative social discounting models described above to participants' gain and loss data and tested a more general hyperbolic equation which included a power function parameter to account for potential sensitivity differences between discounted and undiscounted reward values (6). The hyperbolic social discounting equation with a power function is as follows:

$$\begin{array}{l} v=\;\frac{V}{1+kN^{S}}\; \end{array}$$

where *v, V, k* and *N* are the same as the previous three equations, and *s* represents sensitivity differences between discounted (*v*) and undiscounted (*V*) reward values (i.e., *v*/*V*) for social distance (*N*). When *s* = 1, the power function equation is equivalent to the hyperbolic social discounting equation.

These functions were used to explore cultural differences in Study 2 with participants from either Japan or Germany. The hyperbolic equation including this sensitivity parameter fit the data best for all participants' gain data and Japanese participants' loss data. However, the *q*-exponential equation best fit German participants' loss data. Thus, in both the Takahashi (3) and Ishii and Eisen (5) studies, there was a difference in which model best fit social discounting for losses. Unlike the results of Takahashi, Japanese participants showed the sign effect whereby participants were less likely to discount losses (*k* = 0.09) than gains (*k* = 0.45) as social distance increased. Like the Japanese participants in Takahashi (3), German participants did not show a sign effect. Lastly, Japanese participants discounted gains more as a function of social distance, relative to German participants. No difference was found for socially discounting losses between the two samples.

As shown in the Ishii and Eisen (5) study, researchers have also begun to explore cross-cultural social discounting differences in addition to which equation best fits the obtained data. However, unlike Takahashi (3) and Ishii and Eisen (5), these cross-cultural comparisons have typically given participants the option of either keeping the reward entirely for themselves or the option of equally rewarding another individual [e.g., both receive $75; (1)]. This procedure is more consistent with the notion of sharing, where steep social discounting indicates selfishness, or less sharing. For example, Ito et al. (7) measured group social discounting in both Japanese and United States (US) samples. In this variation of the social discounting task, participants were presented with 30 questions with the option to either (A) keep a monetary reward of varying quantity for themselves or (B) share a specified monetary reward of $1,300 collectively between the individual and a group of varying size and familial status. Discounting rates were lower for both the Japanese and US samples under the relative condition than the stranger condition. However, contrary to their hypothesis, Japanese participants were found to discount more steeply (e.g., choose to share less often) than US participants, similar to Ishii and Eisen (5).

Two additional studies have tested whether participants would either keep all of the money for themselves or split a smaller amount with another person at varying social distances. Strombach et al. (8) compared social discounting between German and Chinese samples. For each social distance, participants were given the option to (A) keep a monetary reward for themselves, or (B) share a reward with the individual at the specified social distance. While both samples showed a decrease in foregoing reward as the social distance increased, Chinese participants were less generous at close social distances, but more generous at farther social distances in comparison to the German participants.

Most recently, Romanowich and Igaki (9) compared social discounting between a Japanese and a US sample. Like Strombach et al. (8), participants were presented with the option to either (A) keep a monetary reward for themselves, or (B) share a reward with the individual at the specified social distance. The task also compared social discounting on a low reward magnitude ($150) and high reward magnitude ($1,500, all amounts multiplied by 10). While no between-culture differences were found in the low reward magnitude condition, US participants shared significantly more than Japanese participants during the high reward magnitude condition, similar to Ito et al. (7) and Ishii and Eisen (5). That is, only Japanese participants showed a magnitude effect by discounting more as the sharable reward magnitude increased.

Social discounting has been linked to important health behaviors, such as addiction. Some of these relationships parallel those found with delay discounting [for review, see (10)]. For example, Bradstreet et al. (11) showed that social discounting was a significant predictor for smoking status (smoker vs. non-smoker), and that smokers shared significantly less than non-smokers. A similar trend was found by Romanowich and Igaki (9), and has also been found in methamphetamine users (12). Additionally, underweight individuals, as measured by body mass index (BMI) seemed to share less than normal weight and obese individuals (13). However, the sample size for underweight individuals was small, relative to the other groups, and these results should be interpreted with caution. In general, these health behavior results are consistent with theories that view addiction as extended temporal and social patterns, which can become problematic when an individual's patterns are too selfish for both themselves and those around them (14). Thus, accurately quantifying social discounting gains and losses can lead to a better understanding of whether social discounting rate can be used as a prospective measure of behavior dysfunction and/or a dependent variable for health treatment outcomes, similar to delay discounting (10).

Based on the social discounting studies by Takahashi (3) and Ishii and Eisen (5), the quantitative discounting functions appear to differentially fit social discounting data for losses and gains. Thus, the first goal of this study was to systematically replicate Takahashi (3) and Ishii and Eisen (5) with a US participant sample to determine whether different social discounting functions fit gains and losses in the same way as these previous two studies. The *q*-exponential equation had the best fit for losses in two of the three groups tested so far [Japanese—(3); Germans—(5)]. We hypothesized that US participants would be consistent with this^1^. Second, we compared absolute differences between gains and losses for each study and the current US participants. All previous studies show that Japanese participants discount social gains more steeply than non-Japanese participants (5, 7, 9). Thus, we hypothesized that US participants would discount gains less than previous Japanese participants, but that there would be no difference in discounted social losses, similar to Ishii and Eisen. Lastly, we tested whether US participants would show a sign effect. Only Japanese participants in Ishii and Eisen (5) have shown a social discounting sign effect in the two previous studies. We hypothesized that US participants, like German participants, would not show the sign effect.

## Methodology

### Participants

Forty volunteers (19 female and 21 male; M age = 19; age range = 18–23) were recruited from Introduction to Psychology courses at the University of Texas at San Antonio (UTSA) using an online subject pool management system. Participants received course credit for participating in the study. Only two participants self-reported smoking cigarettes. This study was approved by the Institutional Review Board (IRB) at UTSA, and all participants were informed of their rights as participants prior to starting the study.

### Procedures

The current study sought to systematically replicate Takahashi (3) and Ishii and Eisen (5), which were both based on (6), within a US sample. The original Japanese version of Takahashi (3) was back-translated (15) by a native Japanese speaker with a high-level of English proficiency. Research assistants interviewed each participant individually with participants seated across a table, facing the researcher throughout the experiment. Participants completed a brief demographics survey, the social discounting gains task, and the social discounting loss task, in that order.

Prior to beginning each social discounting task, participants were provided with both verbal and written instructions:

“*The following experiment asks you to imagine that you have made a list of the 100 people closest to you in the world ranging from your dearest friend or relative at position #1 to a mere acquaintance at #100. The person at number one would be someone you know well and is your closest friend or relative. The person at #100 might be someone you recognize and encounter, but perhaps you may not even know their name. You do not have to physically create the list–just imagine that you have done so.”*

The gains and loss questionnaire format was shown with two options: (A) they receive (are forced to pay) an amount of money themselves, or (B) they let a person at a particular social distance receive (pay) a certain amount. Participants practiced by indicating whom the money would go to by marking an “X” beside their chosen response A (themselves) or B (someone else). For example, participants could choose either (A) $80 for themselves or (B) giving $65 to the person at the social distance ranked 33. After completing the practice question, participants were reminded to very carefully read and focus on each sequential box as the monetary values and scenarios would vary throughout the study. Participants were advised that, although all monetary values within the task were hypothetical, the amounts should be treated as if the money was real.

### Measures

#### Gains

The gains condition consisted of two blocks: one with descending amounts for the participant alone, and one with ascending amounts for the participant alone. During the descending block, the choice for a gain by the participant alone consisted of nine amounts decreasing by $10 from $85 to $5. Participants first completed the descending condition, then completed the ascending block, which began with a $5 gain and ended with an $85 gain, increasing by $10 for each subsequent choice. The choice to allocate the money to the other person was a constant $75 for both blocks.

On each subsequent page, the choice to allocate money to the other person consisted of one of seven social distances: 1, 2, 5, 10, 20, 50, and 100, presented in ascending order. Participants responded to the descending and ascending gain blocks for all seven social distances. In total, participants made 126 choices during the gains condition: 63 during the descending gain block and 63 during the ascending gain block.

#### Losses

Similar to the gain condition, participants made an additional 126 choices about taking a monetary loss or allocating that loss to another person. During the decreasing block, the choice for a loss by the participant alone consisted of nine amounts decreasing by $10 from $85 to $5. Participants first completed the descending condition, then completed the ascending block, which began with a $5 loss and ended with an $85 loss, with losses increasing by $10 for each subsequent choice. The choice to allocate the loss to the other person was a constant $75 for both blocks.

On each subsequent page, the choice to allocate losses to the other person consisted of one of seven social distances: 1, 2, 5, 10, 20, 50, and 100, presented in ascending order. Participants responded to the descending and ascending loss blocks for all seven social distances. In total, participants made 126 choices during the loss condition: 63 during the descending loss block and 63 during the ascending loss block.

#### Crossover Points

Based on Takahashi (3), cross-over points were calculated for each participant for each social discounting point and served as an estimate of the average of the last selfish (Choice A) act and the first generous (Choice B) act in the gains condition. An indifference point was computed by averaging the crossover points from both ascending and descending conditions. Indifference points are defined as a subjective value of reward that a person at varying social distances received (i.e., *v* in equations 1–4). In contrast to the gain conditions, participants in the loss condition were instructed that either they or one of their respective imagined social connections would be forced to lose a particular amount of money. From this perspective, the generous act would be to take the loss (Choice A), whereas the selfish act would be to allocate the loss to others (Choice B).

#### Analyses

To test the first two hypotheses, participants' median gain and loss data were fit to each of the four social discounting equations described in the Introduction. This resulted in *k, v*, and either *q*- or *s*-values for the *q*-exponential or hyperbolic power function discounting equations. For the hyperbolic and exponential functions, both *V* and *k* were estimated from the behavioral data. In addition, for the *q*-exponential and hyperbolic power functions either *q* or *s* were estimated from behavioral data, respectively. Individual data was also fit to each of the four equations. Fit for each equation was estimated using the AIC statistic, which calculates tradeoffs between overfitting and poor fitting models. Poor fitting models result in larger AIC values. To test the third hypothesis, area-under-the-curve (AUC) was calculated for each participant's gain and loss data using the method described by Borges et al. (16). Specifically, AUC_ord_ was calculated which is an ordinal scaling transformation for social distance. This method equates contributions to overall AUC for both small and large social distances. To compare absolute AUCs with Ishii and Eisen (5), the non-scaled AUC was calculated [i.e., (17)].

## Results

**Figure 1:**
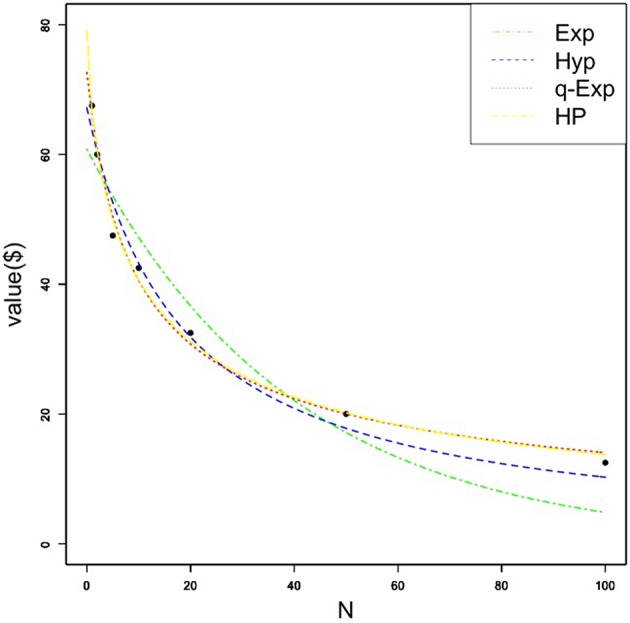
Median amount of money (in US dollars) forgone as a function of social distance for the gain condition. Each quantitative social discounting function was fit to the estimated parameters using nonlinear regression. Exp, Hyp, q-Exp, and HP stand for the exponential, hyperbolic, *q*-exponential, and hyperbolic power functions, respectively.

First, we examined the fit of the four functions for gains and losses for group data: the exponential function, hyperbolic function, *q*-exponential function, and hyperbolic power function. Since previous studies used either medians [e.g., (3)] or means [e.g., (5)] for fitting equations, the functions were fit using both the median (Table 1) and the mean (Table 2). The tables show the obtained *k, v, q* or *s* values and corresponding standard errors (SE) for each function across both gains and losses. For gains, the hyperbolic power function best fit group data relative to the other three social discounting equations, consistent with Ishii and Eisen (5), using either the median (AIC = 32.84) or the mean (AIC = 26.65). A Kolmogorov-Smirnov test was conducted on individual *k*-values for each gain function and showed that none of the *k*-values were normally distributed (*D* = 0.27-0.52; all *p*'s < 0.01). Thus, Figure 1 shows each of the four discounting functions fit to the median gain data. Except for the exponential function, each function fit the obtained data well. For individual participant gain data, the hyperbolic power function best fit 14 participants' data, followed by the *q*-exponential (*n* = 10), hyperbolic (9), and exponential functions (7). The median *s* value for US participants was also less than 1, similar to both Japanese and German samples. However, the absolute *s* value for US participants (*s* = 0.70) was more similar to the German (*s* = 0.66), relative to the Japanese (*s* = 0.48) sample. For losses, the *q*-exponential best fit the data using the median (AIC = 40.12), consistent with Takahashi (3) and German participants in Ishii and Eisen (5); however, when using the mean, the hyperbolic power function had the best fit (AIC = 13.59).

**Table 1:** Median discount parameters for each of the four social discounting equations.

	**Exponential**	**Hyperbolic**	***q*-Exponential**	**Hyperbolic power**
**Gains**				
AIC	49.853	39.870	35.421	32.838
*k* (SE)	0.025 (0.006)	0.055 (0.009)	0.104 (0.031)	0.188 (0.072)
*V* (SE)	60.806 (4.187)	67.189 (2.78)	72.646 (3.531)	79.052 (6.124)
*q* or *s* (SE)	–	–	−0.819 (0.335)	0.701 (0.083)
**Loss**				
AIC	50.745	40.217	40.116	41.218
*k* (SE)	0.02 (0.004)	0.04 (0.005)	0.051 (0.015)	0.06 (0.035)
*V* (SE)	73.508 (4.217)	79.217 (2.561)	81.07 (3.36)	81.799 (5.098)
*q* or *s* (SE)	–	–	−0.417 (0.407)	0.891 (0.142)

*AIC, Akaike Information Criterion (Smaller AIC indicates better fit); k, constant measuring degree of discounting; V, undiscounted value of reward; q, degree of “social inconsistency” from exponential discounting (q-exponential function only); s, sensitivity differences between discounted and undiscounted reward values (hyperbolic power function only)*.

**Table 2:** Mean discount parameters for each of the four social discounting equations.

	**Exponential**	**Hyperbolic**	***q*-exponential**	**Hyperbolic power**
**Gains**				
AIC	44.408	33.709	29.223	26.650
*k* (SE)	0.016 (0.003)	0.031 (0.003)	0.046 (0.009)	0.077 (0.021)
*V* (SE)	57.412 (2.566)	61.19 (1.496)	63.538 (1.537)	65.914 (2.142)
*q* or *s* (SE)	–	–	−0.686 (0.282)	0.779 (0.063)
**Loss**				
AIC	44.631	32.460	15.793	13.585
*k* (SE)	0.013 (0.002)	0.024 (0.002)	0.036 (0.002)	0.058 (0.006)
*V* (SE)	66.282 (2.531)	69.738 (1.285)	72.088 (0.547)	74.027 (0.74)
*q* or *s* (SE)	–	–	−0.767 (0.11)	0.791 (0.023)

*Abbreviations: see Table 1*.

**Figure 2:**
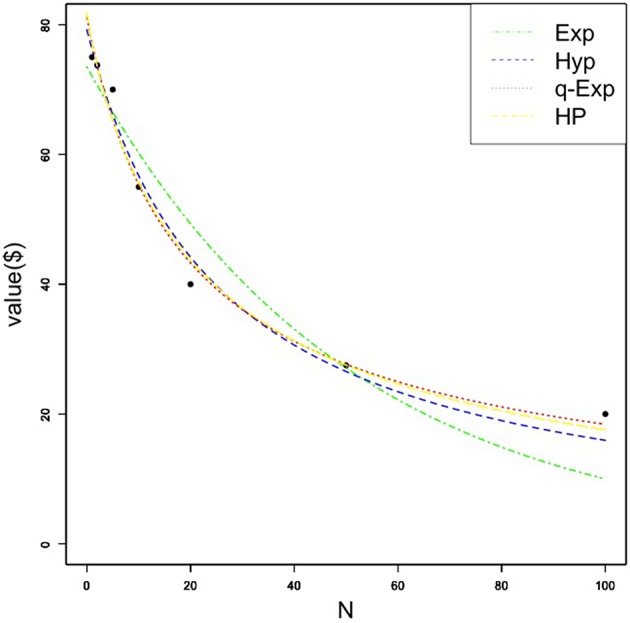
Median amount of money (in US dollars) forgone as a function of social distance for the loss condition. Each quantitative social discounting function was fit to the estimated parameters using nonlinear regression. Exp, Hyp, q-Exp, and HP stand for the exponential, hyperbolic, *q*-exponential, and hyperbolic power functions, respectively.

A Kolmogorov-Smirnov test was conducted on individual *k*-values for each loss function and again showed that none of the *k*-values were normally distributed (*D* = 0.49-0.54; all *p*'s < 0.01). Figure 2 shows each of the four discounting functions fit to the median loss data. Similar to gain data, only the exponential function did not fit the obtained data. For individual participant loss data, the hyperbolic function best fit 13 participants' data, followed by the hyperbolic power (*n* = 11), exponential (10), and *q*-exponential functions (6). The *q*-value for US participants was negative, suggesting a degree of interpersonal inconsistency for social choices. However, this *q*-value for losses was larger than the previous social discounting for losses (*q* < −0.73) with Japanese and German participants.

For absolute social discounting rate comparisons, we compared the *k* values for the best fitting quantitative social discounting equation for each participant group across both gains and losses. However, no statistical comparison could be made as *k*-value variance was not reported in either Takahashi (3) or Ishii and Eisen (5). The hyperbolic power function best fit gain data for the current US sample (see Table 1) and both Japanese and German participants in Ishii and Eisen (5). Table 2 shows the US sample's mean *k*-value (*k* = 0.08) was larger than German participants (*k* = 0.05), but smaller than Japanese participants (*k* = 0.10) from Ishii and Eisen. Thus, consistent with all previous studies (5, 7, 9), US participants socially discounted less steeply than Japanese participants.

For losses, the *q*-exponential equation best fit the median data for Japanese participants in Takahashi (3) and the mean data for German participants in Ishii and Eisen (5). However, for Japanese participants in Ishii and Eisen, the hyperbolic power function best fit the mean loss data. As shown in Table 2, the hyperbolic power function also best fit US participants' mean loss data. When comparing across groups with the hyperbolic power function, US participants' mean *k*-value (*k* = 0.06) was larger than both German (*k* = 0.05) and Japanese (*k* = 0.04) participants (i.e., more social discounting). However, this trend was reversed when comparisons were based on the *q*-exponential equation. Here, US participants' mean *k*-values were the lowest (*k* = 0.04) relative to Japanese (*k* = 0.09) and German (*k* = 0.10) participants. Comparisons with Takahashi (3) showed that US participants' median *q*-exponential *k*-value for losses was lower (*k* = 0.05) than for Japanese participants (*k* = 0.06).

Second, we compared social discounting for gains and losses between our sample of US participants and other two studies. Relative to Ishii and Eisen (5), the current US participants' mean non-scaled AUC for gains (μ = 0.34; σ = 0.20) was more similar to German participants (μ = 0.36; σ = 0.20), than Japanese participants (μ = 0.29; σ = 0.23). A one-way ANOVA using the Ishii and Eisen (5) summary data showed a significant group difference, *F*_(2, 257)_ = 3.03, *p* < 0.05. However, Tukey HSD *post-hoc* tests only showed differences between German and Japanese participants, as originally reported in Ishii and Eisen (5). In terms of mean non-scaled AUC for losses, US participants discounted losses less steeply (μ = 0.42; σ = 0.20), relative to both German (μ = 0.36; σ = 0.20) and Japanese (μ = 0.36; σ = 0.25) participants. A one-way ANOVA using the summary data did not show a significant group difference, *F*_(2, 257)_ = 1.21, *p* = 0.30.

**Figure 3:**
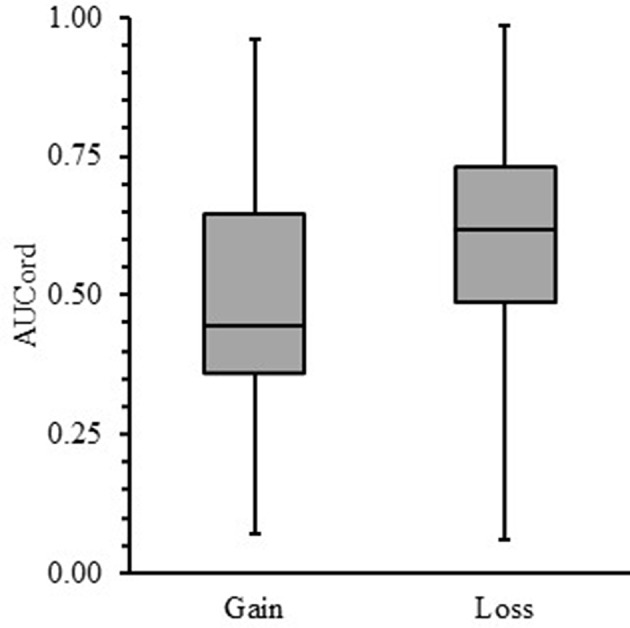
Box-and-whisker plots for area-under-the-curve (AUC_ord_) as a function of social discounting gains and losses. The end of each box demarcates the upper and lower quartiles, whereas the median is demarcated by the horizontal line bisecting each box. Vertical lines above and below each box represent the range of obtained data.

Lastly, we tested whether US participants would show a sign effect. A Shapiro-Wilk test showed that both gains (*Mdn* = 0.45; *W* = 0.96; *p* = 0.13) and losses (*Mdn* = 0.62; *W* = 0.98; *p* = 0.52) were normally distributed. Therefore, a paired *t*-test was used to determine whether US participants showed a sign effect. Figure 3 shows that the average AUC_ord_ for losses was significantly larger than for gains [*t*_(39)_ = 3.17, *p* < 0.01, *d* = 0.50]. Thus, US participants showed a sign effect by discounting losses less steeply as a function of social distance, relative to equal gains.

## Discussion

The present study was a systematic replication of Takahashi (3) and Ishii and Eisen (5) testing which quantitative social discounting function best fit gains and losses for a US sample. The hyperbolic power function best fit gain data, consistent with Ishii and Eisen (5). The *q*-exponential function best fit loss *median* data (Table 1), whereas the hyperbolic power function best fit the *mean* loss data (Table 2). Differential quantitative fits between gains and losses (for median data) were consistent with German participants from Ishii and Eisen and Japanese participants from Takahashi (3), and supported the first hypothesis. However, there were no statistically significant differences in overall social discounting for either gains or losses between the US sample and participants from Ishii and Eisen (5). Thus, our second hypothesis was only partially supported, whereby there was no difference in social discounting for losses across groups. Lastly, our third hypothesis was not supported. US participants showed a robust social discounting sign effect (Figure 3) in contrast to German participants.

Similar to previous studies, the best fitting quantitative social discounting model differed for median gains and losses. When participants socially discount losses, they are choosing whether to allocate a financial burden with another person at a certain social distance. If social discounting for gains means giving up a potential positive reinforcer, social discounting for losses means giving up a potential negative punisher. From this point of view it seems plausible that the discounting functions would be different between social gains and losses. The hyperbolic power function has consistently been shown to best fit social discounting gains data across cultures, whereas social discounting loss data is typically best fit by the *q*-exponential function. This inconsistency between which quantitative social discounting equation best captures participants' data suggests a cross-cultural asymmetry in behavioral processes for equal gains and losses. This asymmetry is not new, as prospect theory (18) was proposed to account for different behaviors when equal gains and losses were at stake. Estle et al. (4) showed changing asymmetries between gain and losses depending on discounting type (delay or probability) and reward magnitude. More specifically, the sign effect was magnified for delayed rewards at relatively small reward magnitudes, whereas for probabilistic rewards at relatively large reward magnitudes increased the sign effect. Social discounting experiments have repeatedly demonstrated that participants discount more steeply as magnitude increases (6, 9, 19), which is similar to probability discounting. No social discounting experiment has compared gains and losses as a function of reward magnitude. However, if there is consistency between previous social and probability discounting results, the sign effect should increase as magnitudes increase during social discounting tasks.

More pertinent to the current study is whether the best fitting equation would remain constant as magnitude changes across different cultures. The *s* value in the hyperbolic power function represents a psychological scaling parameter, in this case for undiscounted value (*V*) and social distance (*k*). In all previous cross-cultural social discounting studies using Rachlin and Jones' (6) methodology, the mean and/or median *s* value has been < 1.0, while the median *s* value for Rachlin and Jones' participants was 1.03. *s* values < 1.0 suggest that subjective value declines more rapidly for smaller social distances (i.e., 1, 2, and 5), and less rapidly for larger social distances. For delay discounting gains, *s* values < 1.0 are the norm (20). In addition, a few studies have shown that *s* values for delay discounting losses are < 1.0 (21) and relatively consistent across increasing loss magnitudes (4, 22). If social discounting is consistent with delay discounting findings *s* values for gains, and possibly losses, should be invariant as reward magnitude increases. Ultimately, this is an empirical question that needs additional research across a range of magnitudes and cultures.

There were no statistically significant differences in overall social discounting for gains between the US sample and participants from Ishii and Eisen (5). There are a few possible reasons for the failure to find differences between cultures. First, sample sizes were different between US participants (*n* = 40) and those from Ishii and Eisen (*n* > 100). A larger US sample size may have provided more adequate power to show group differences. However, needing sample sizes > 100 to show a statistically significant group difference suggests a small effect size. Instead, we believe that an alternate explanation based on the magnitude effect is more tenable. Romanowich and Igaki (9) showed differential magnitude sensitivity between US and Japanese participants. However, Romanowich and Igaki showed no group difference at the magnitude used in the current study (~75–85 USD), and those by Takahashi (3) and Ishii and Eisen (5). For US participants, Rachlin and Jones (6) demonstrated a between-subjects social discounting magnitude effect by using three reward magnitudes ($7.50, $75, and $75,000). No studies have tested the magnitude effect with German participants. Thus, without varying reward magnitude it is still unknown whether a group difference for social discounting gains (or losses) truly exists. At present, we can conclude that *at this reward magnitude* there are no cross-cultural differences for social discounting gains or losses between US participants and German and Japanese participants. Future studies will need to clarify whether the sign and magnitude effects for social discounting interact with culture, and how those effects can be adequately and reliably quantified.

US participants showed a robust sign effect for social discounting, similar to Japanese participants in Ishii and Eisen (5), but contrary to German participants in that same study. In terms of psychological mechanisms that might account for cross-cultural differences, Ishii and Eisen (5) used harmony-seeking [i.e., (23)] as a mediating variable to explain social discounting gain differences between German and Japanese participants. The construct of harmony-seeking was positively correlated to the obtained AUCs and showed a mediating role between AUC and culture in a multiple regression analysis. However, this construct also had questionable to poor reliability for both Japanese (α = 0.66) and German (α = 0.53) participants, respectively. Thus, it is still uncertain whether the harmony-seeking construct can explain cross-cultural social discounting differences, especially as they relate to within-culture differences between social gains and losses.

Except for median social discounting losses (see Table 1), either the *q*-exponential or the hyperbolic power function had the lowest AIC values. In the case of median social discounting losses, all functions that included some aspect of hyperbolic discounting produced favorable fits (*q* values ≤ 0 suggest hyperbolic instead of exponential discounting). However, even if data fits the *q*-exponential and hyperbolic power functions similarly, there are psychological differences in the *q* and *s* parameters. *q* is a behavioral parameter indicating how much a participant's actual social discounting behavior deviates from exponential social discounting behavior. *s* is a psychophysical parameter indicating non-linear distortion in the perceived social distance for hyperbolic social discounting. Moreover, *q* is also *indirectly* related to psychophysical distortion in social distance perception, as is the case with time discounting with non-linear time perception [see (24)]. Therefore, it is important for future cross-cultural social discounting studies to specifically examine the role of social distance perception. Examining social distance perception will help to elucidate the psychological mechanisms underlying the observed difference in fit for different social discounting functions for gain and loss across different cultures (see Results section).

The present results also have implications for important health behaviors, such as addiction. No previous studies examining the relationship between social discounting and health behaviors have compared best fitting quantitative models to gains and losses. Accurately quantifying participants' social discounting behavior can help illuminate relationships with these health behaviors. The more accurate this relationship is, the more likely that the social discounting task alone can be used as a prospective measure for health behavior risk, and/or a dependent variable for treatment. For example, individuals that discount very steeply during a social discounting task may be at greater risk for drug use (11, 12) and/or eating disorders (13). It is likely that individuals with addictions socially discounting in a more hyperbolic manner (i.e., large decrease in resource allocation at small social distances), similar to delay discounting. Likewise, deviations in an individual's quantitative fit for a social discounting task may also be indicative of disordered health behaviors. This may signal abnormal social behavior patterns [i.e., hyper-selfishness; (14)] that place the individual at additional risk for developing health behavior problems. Future work in this area promises to be impactful given the significant relationship between delay and social discounting (13, 25). However, as mentioned above, the exact mechanism controlling social discounting deviations need to be better understood to make these potential associations more precise and meaningful.

Although we attempted to replicate the procedural details from Takahashi (3), other methodological differences such as the context where the social discounting task was administered may be contributing factors to observed differences between all three studies. The present study was methodologically more similar to Takahashi (3) than Ishii and Eisen (5), whereby we collected data by conducting one-on-one interviews with each participant. Ishii and Eisen (5) also utilized paper-and-pencil surveys for data collection, but participants completed the social discounting task individually. The presence (or absence) of an experimenter or other participants may differentially influence the likelihood of sharing across different cultures (26). Additional research is needed on these potential observer effects in cross-cultural discounting research. Likewise, social discounting task presentation was held constant, whereas Ishii and Eisen (5) counterbalanced between gain and loss tasks. Task presentation order may have changed social discounting for individuals that were still learning about what was being asked after multiple iterations of each task. Like potential observer effects, more research should target whether individuals produce different social discounting outcomes with more social discounting task experience.

Lastly, these three studies were conducted at least 6 years apart. It is unknown how stable social discounting is within a population. Thus, many other factors outside culture could have contributed to social discounting differences. For example, economic changes such as an unstable currency (27) have been shown to influence delay discounting. While it is unknown how currency stability effects social discounting, future studies should control for economic variables such a relative buying power, consumer price index, and/or each country's gross domestic product.

### Limitations and Future Directions

Outside of methodological and temporal differences, the current study sample was limited to undergraduate students at a university located in the southwest US. Given the diversity of the US, these results should not be generalized without caution to other regions of the US. In addition, demographic variables such as participant income, nationality, and country of origin, were not controlled for the present study. However, university students have been shown to vary as much as the general population in behavior-based phenomena (28). Although participants were advised to behave as if their choices involved real money, the social discounting measure was still based on hypothetical outcomes, not a real-world outcome. Therefore, additional non-hypothetical scenarios may provide insight beyond self-report to capture how participants across different cultures would behave during the social discounting task. Currently, studies exploring whether hypothetical social discounting outcomes are similar to real outcomes have only measured within-culture similarities and differences (29, 30), with different results. Thus, future cross-cultural social discounting research should further examine real vs. hypothetical outcomes. As a direct replication for Takahashi (3), the current sample size suffices for such a direct comparison. However, when examining multiple groups/cultures and phenomena (i.e., gains vs. losses; magnitude effects) increased sample sizes will be necessary for future research to reach acceptable statistical power.

The current results suggest a need to further evaluate the best fit of both the *q*-exponential and hyperbolic power function in different populations across social discounting gains and losses. It is also valuable to include participants with important health behaviors such as smoking and illicit drug use. Future studies should begin to conceptualize social discounting rate as a potential marker for dysfunctional behavior and/or as a dependent measure for treatment outcomes, similar to delay discounting. In addition, comparing social discounting results between German, Japanese, and US participants has demonstrated cultural similarities for the best quantitative data fit, and differences for sign effect prevalence. Previous quantitative research comparing temporal and probability discounting across sign and magnitude (4, 31) can serve as both a guide and comparison for future cross-cultural social discounting research.

## Data Availability Statement

The raw data supporting the conclusions of this manuscript will be made available by the authors, without undue reservation, to any qualified researcher.

## Ethics Statement

This study was carried out in accordance with the recommendations of the Institutional Review Board (IRB) at UTSA with written informed consent from all subjects. All subjects gave written informed consent in accordance with the Declaration of Helsinki. The protocol was approved by the IRB at UTSA.

## Author Contributions

PR conceived and planned the systematic replication based on original research by TT. TC carried out the study with assistance from master's level graduate students. TT, PR, and TK contributed to analyzing the raw data and interpretation of results. SS took the lead on manuscript writing and contributing to every section. TC contributed to the method and discussion section. PR contributed write up of the results and supervised with edits throughout. TK and TT contributed to portions of the introduction and discussion sections. SS, TC, TK, TT, and PR provided edits and added content throughout the manuscript.

### Conflict of Interest

The authors declare that the research was conducted in the absence of any commercial or financial relationships that could be construed as a potential conflict of interest.

## Footnotes

^1^Takahashi (3) did not compare the *q*-exponential function with the hyperbolic power function. Therefore, it is unknown whether the hyperbolic power function would have fit Japanese participants' social discounting losses better than the *q*-exponential function during this study.
